# A Rare Case of Regorafenib-Induced ST-Elevation Myocardial Infarction

**DOI:** 10.7759/cureus.39779

**Published:** 2023-05-31

**Authors:** Sadichhya Karki, Vaishali Deenadayalan, Prajwal Shrestha, Samriddh Dhungel, Aviral Vij

**Affiliations:** 1 Internal Medicine, John H. Stroger, Jr. Hospital of Cook County, Chicago, USA; 2 Medicine, John H. Stroger, Jr. Hospital of Cook County, Chicago, USA; 3 Cardiology, Cook County Health, Chicago, USA

**Keywords:** st-elevation myocardial infarction, small molecule tyrosine kinase inhibitors, tyrosine kinase inhibitors, colorectal cancer, stemi, regorafenib

## Abstract

Regorafenib is an oral multi-kinase inhibitor that is used in the treatment of chemotherapy-resistant metastatic colorectal carcinoma. However, multi-kinase inhibitors have been known to cause cardiac side effects, most notably hypertension. Myocardial ischemia is a very extraordinary adverse effect of regorafenib. Our patient was a 74-year-old gentleman with stage IVa colon cancer who underwent a right colectomy with end ileostomy and was on cycle two of regorafenib during the presentation. He came in with acute onset chest pain that was intermittent, non-exertional, and radiating to the back. His left heart catheterization did not reveal any atherosclerotic lesions, and his ST-elevation myocardial infarction (STEMI) was deemed an extremely rare adverse event from regorafenib. We are herewith reporting a case of regorafenib-induced STEMI.

## Introduction

Regorafenib is a small-molecule multi-kinase inhibitor that has shown significant efficacy in the treatment of various malignancies, including metastatic colorectal carcinoma (mCRC) [1], advanced gastrointestinal stromal tumors (GIST) [2], and hepatocellular carcinoma (HCC) [3]. However, like many targeted therapies, regorafenib is associated with a range of adverse effects, including cardiotoxicity. Cardiotoxicity refers to the potential for drugs to cause damage to the cardiovascular system, leading to functional impairment and potentially life-threatening complications.

The incidence and clinical manifestations of regorafenib-induced cardiotoxicity have gained increasing attention in recent years. Several case reports and small studies have documented cardiac adverse events associated with regorafenib use, including myocardial ischemia, heart failure, arrhythmias, and sudden cardiac death. However, the underlying mechanisms remain poorly understood. We are reporting a case of acute chest pain in a patient on regorafenib therapy. Additionally, we will discuss the challenges in the early detection and management of cardiotoxicity associated with regorafenib, highlighting the need for further research in this field.

## Case presentation

A 74-year-old gentleman with stage IVa colon cancer status post right colectomy with end ileostomy, currently on cycle two of regorafenib, presented to the emergency department with acute onset, intermittent, non-exertional, midsternal chest pain, radiating to the back every few hours for one week. It was associated with nausea and diaphoresis without any exacerbating or relieving factors. At presentation, he was afebrile and hypertensive to 153/76 mmHg, with a heart rate of 82 beats/minute, respiratory rate of 20/minute, and saturating at 98% on room air. Physical examination revealed a single S1 and S2 heart sound without gallop or murmur, and no rales or wheezing were auscultated. His laboratory values on admission are presented in Table 1.

**Table 1:** Laboratory values on admission WBC: white blood cells; Hb: hemoglobin; Na: sodium; K: potassium; BUN: blood urea nitrogen; LDL: low-density lipoprotein; HbA1c: glycosylated hemoglobin.

Test	Result	Reference range
WBC	8.9	4.0-11.0 k/uL
Hb	10.9	12.0-16.0 gm/dL
Platelets	234	150-450 k/uL
Na	138	135-145 mEq/L
K	4.0	3.5-5.0 mEq/L
Bicarbonate	26	22-30 mEq/L
Glucose	98	70-100 mg/dL
BUN	17	7-20 mg/dL
Creatinine	0.8	0.6-1.3 mg/dL
Albumin	3.8	3.4-5.0 g/dL
LDL	67	<100 mg/dL
HbA1c	4.9	<5.7%

**Figure 1:**
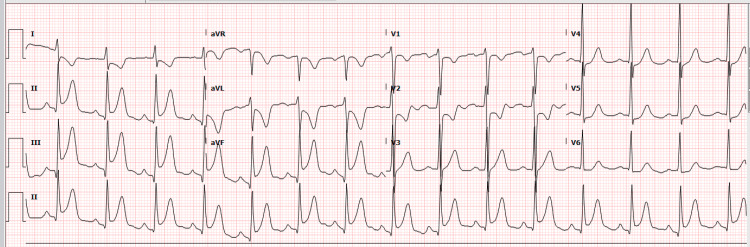
Electrocardiogram showing ST elevation in inferior leads

Chest radiograph showed a known small pulmonary nodule without any acute cardiopulmonary process. Electrocardiogram (EKG) was significant for ST elevation in inferior leads with dynamic T wave changes (Figure 1). Troponin obtained was 0.18 (reference: <0.019 ng/ml) initially, which increased to 0.267 and peaked at 0.354 within eight hours of presentation.

**Figure 2:**
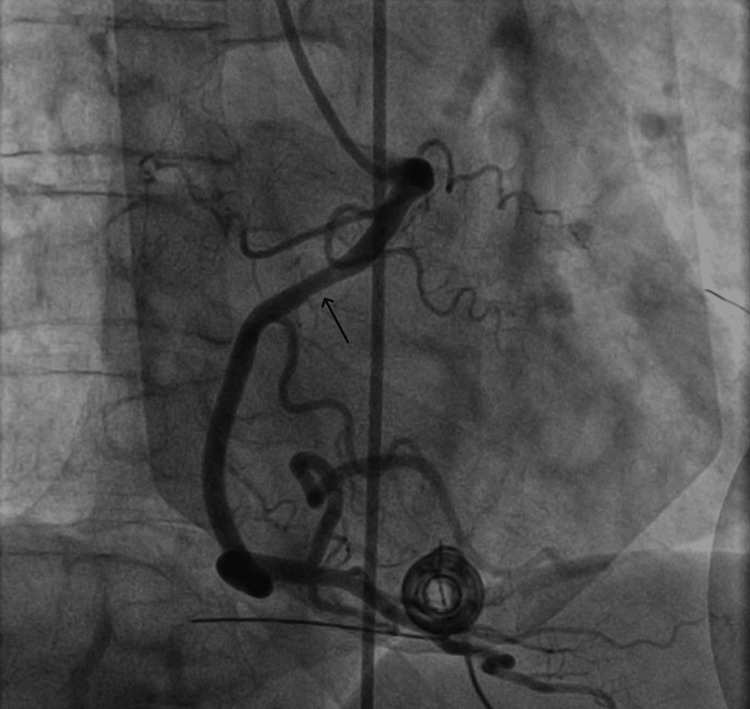
Coronary angiogram showing filling defect in proximal right coronary artery

**Figure 3:**
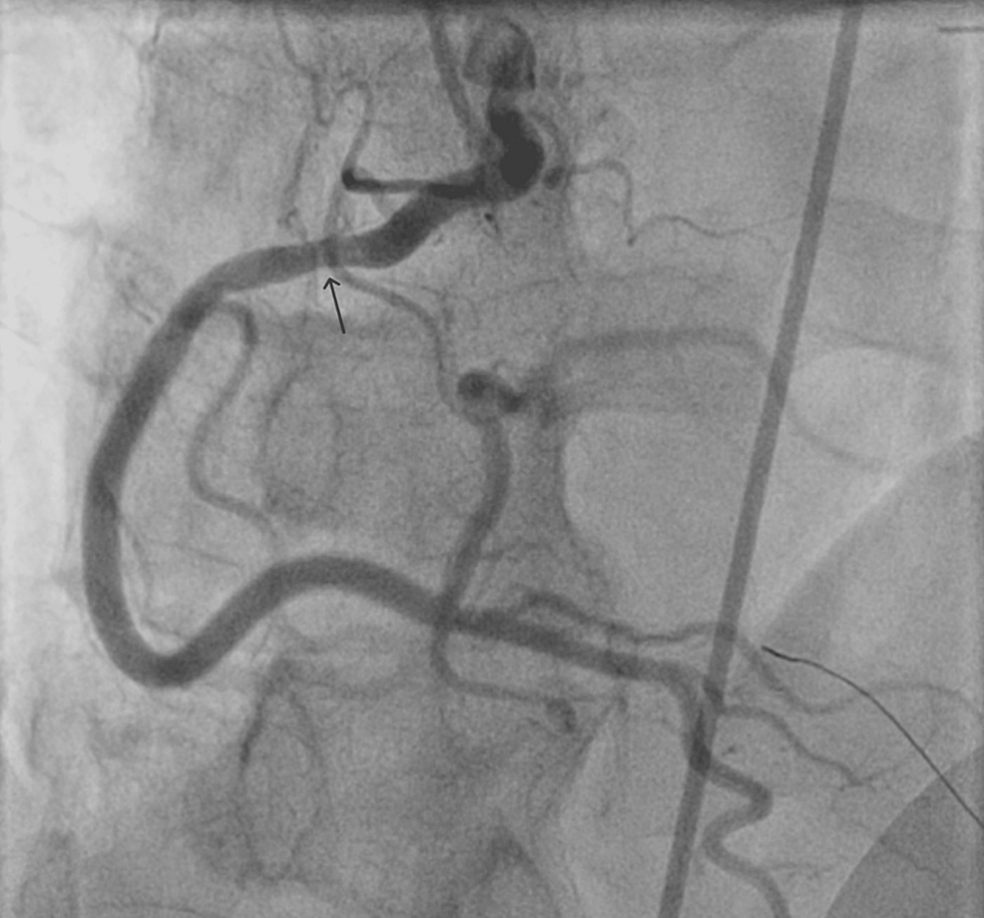
Coronary angiogram showing hazy-appearing proximal right coronary artery with concern for a filling defect

The patient was loaded with aspirin and was taken for an emergent coronary angiography, which demonstrated non-obstructive coronary artery disease but with a filling defect in the proximal right coronary artery (RCA) (Figures 2, 3).

**Figure 4:**
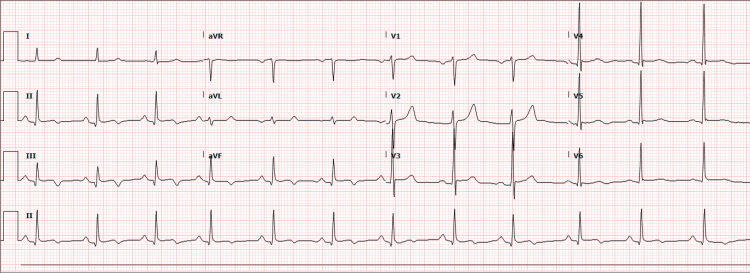
Repeat electrocardiogram showing resolving ST changes in inferior leads. This was obtained within one hour of admission to the coronary care unit

For further understanding, a coronary CT angiogram was obtained, which showed normal coronaries. Post-angiogram, the patient was transferred to the coronary cardiac unit (CCU), and his chest pain resolved. Follow-up EKG within an hour (Figure 4) demonstrated resolving ST-T wave changes. Transthoracic echocardiography showed normal left ventricular function and an ejection fraction of 65% with no regional wall motion abnormality. The patient continued to have high blood pressure.

At this point, we presumed his ST-elevation myocardial infarction (STEMI) presentation was secondary to vasospasm induced by tyrosine kinase inhibitors. The medical oncology team was consulted and after a discussion with the patient and family, a decision was made to hold off on regorafenib. The next day, the patient was discharged on aspirin and beta blockers. During his later follow-ups with the oncologist, he was chest-pain free and wished for other palliative options for his malignancy.

## Discussion

Small molecule tyrosine kinase inhibitors are a new class of targeted therapy that interferes with cell signaling pathways that drive certain malignancies. They include imatinib mesylate, dasatinib, nilotinib, sunitinib, sorafenib, and lapatinib. In recent years, multi-targeted kinase inhibitors have shown attendant benefits by exerting their anti-angiogenesis effect, which not only degrades the microvessels of tumors but also causes liquefactive necrosis of tumor cells [4]. Several trials have acknowledged the survival benefits of regorafenib therapy in patients with metastatic colorectal cancer, GIST, and HCC [5]. Based on these results from multiple studies, regorafenib received approval for treatment of mCRC in patients who have been previously unresponsive to fluoropyrimidine-, oxaliplatin-, and irinotecan-based chemotherapy, anti-vascular endothelial growth factor (VEGF) therapy, and an anti-epidermal growth factor receptor (EGFR) therapy in KRAS wild-type patients [6]. Despite this revolutionary progress, these drugs are found to be associated with various cardiovascular side effects.

As a class effect, these drugs primarily target the vasculature rather than the tumor cells themselves. They have a wide spectrum of toxicities that are unique and may be fatal as well. Regorafenib is a relatively new agent and targets VEGF receptors 1-3, KIT, PDGFR alpha and beta, RET, rGRF 1 and 2, TIE2, DDR2, TrkA, RAF-1, BRAF, and many others. The VEGF receptor and its downstream functions are very important for the integrity of normal endothelial and vascular function [7]. Research on mice has shown the existence of pericyte-endothelial-myocardial coupling and tyrosine kinase inhibitors (sunitinib in this study) can interrupt the function of any cells at various pathways leading to reduced myocardial pericytes, decreased myocardial microvascular density, and ultimately myocardial function and reserve [8].

In our case, the patient was diagnosed with metastatic adenocarcinoma of the colon (KRAS mutant type) when he came to the emergency department in the fall of 2020 for acute bowel obstruction. At that admission, he underwent exploratory laparotomy, right colectomy with the creation of end-ileostomy, and mucous fistula of the transverse colon (Prasad type). He received palliative chemotherapy with FOLFOX (folinic acid, fluorouracil, and oxaliplatin) and bevacizumab, followed by FOLFIRI (folinic acid, fluorouracil, and irinotecan) + bevacizumab; however, his carcinoembryonic antigen (CEA) continued to trend up. Hence, he started regorafenib 160 mg daily every 21 days in December 2022. The plan was to continue four cycles and reassess disease progression.

A study by Kim et al. [9] on 81 patients on nilotinib therapy demonstrated ECG abnormality in 40% of the study population. Among them, 16 patients (20%) developed new changes including rhythm abnormalities (5%), conduction disturbances (1%), ST segment changes (2%), T wave changes in two (2%), and QTc prolongation in nine (11%) patients. Per the CORRECT trial, regorafenib was found to increase the incidence of myocardial ischemia and infarction (1.2% for regorafenib vs. 0.4% for placebo). Most of the side effects occurred early (within one to two cycles) of the therapy and were controlled with the reduction or interruption of regorafenib. Our patient also developed STEMI on the second cycle of his regorafenib.

There is no case report of acute coronary syndrome from regorafenib in the literature search. A meta-analysis by Chen et al. reported hypertension and hemorrhage as the most common cardiovascular events associated with regorafenib [10]. They were speculated to occur as a result of endothelial dysfunction and abnormal nitric oxide metabolism. Our patient also had intermittent hypertension on this presentation, and the maximum recorded blood pressure was 192/109 mmHg. Another postulated mechanism is the inhibition of tyrosine kinases in the cardiac myocytes and mitochondrial damage leading to myocardial ischemia and infarction [11-13]. Hsiao et al. reported a case wherein a patient previously on nilotinib, later switched to regorafenib, had a history of recurrent chest pain, which was associated with paroxysmal atrial fibrillation with a rapid ventricular rate and positive cardiac biomarkers, who also had a similar coronary angiography finding with no significant coronary stenosis as in our patient [14].

The left heart catheterization (LHC) showed a filling defect in proximal RCA, which could be an episode of vasospasm given no evidence of any obstruction by thrombus in coronary CT. This localized finding also correlated with his EKG findings of ST-elevation in inferior leads along with uptrending troponin levels. These features suggested that our patient had transient vasospasm of RCA leading to STEMI presentation.

As regorafenib is approved for use in treatment-refractory metastatic colorectal cancer patients, any adverse effect of treatment on quality of life will outweigh the benefit of treatment. Hence, patients should be closely followed and monitored with standard and validated quality-of-life measurement tools as used in the CORRECT trial. Treatment should be withheld for any severe toxicity of treatment. Our patient had a STEMI after starting regorafenib and needed hospitalization and intervention as well. Hence, regorafenib was stopped, taking into consideration his quality of life.

## Conclusions

With the advent of newer targeted therapies in oncology, the incidence of their systemic side effects is also increasing. Chemotherapy-induced cardiac toxicities have been established as the second cause of death in patients undergoing chemotherapy. This is the first case of STEMI presentation due to regorafenib toxicity in our knowledge. Although myocardial infarction is an uncommon adverse effect of regorafenib, vigilance and early diagnosis are essential for timely intervention in this subpopulation. More studies evaluating the mechanism of cardiotoxicity with regorafenib might help to reduce or mitigate the untoward cardiovascular adverse effects.
